# Nanoparticles overcome adaptive immune resistance and enhance immunotherapy *via* targeting tumor microenvironment in lung cancer

**DOI:** 10.3389/fphar.2023.1130937

**Published:** 2023-03-24

**Authors:** Xin Zhang, Xuemei Wang, Lijian Hou, Zheng Xu, Yu’e Liu, Xueju Wang

**Affiliations:** ^1^ Department of Pathology, China-Japan Union Hospital, Jilin University, Changchun, China; ^2^ School of Medicine, Tongji University Cancer Center, Shanghai Tenth People’s Hospital of Tongji University, Tongji University, Shanghai, China

**Keywords:** nanoparticles improve immunotherapy TME, PD-L1, PD-1, nanoparticles, immunotherapy, resistance

## Abstract

Lung cancer is one of the common malignant cancers worldwide. Immune checkpoint inhibitor (ICI) therapy has improved survival of lung cancer patients. However, ICI therapy leads to adaptive immune resistance and displays resistance to PD-1/PD-L1 blockade in lung cancer, leading to less immune response of lung cancer patients. Tumor microenvironment (TME) is an integral tumor microenvironment, which is involved in immunotherapy resistance. Nanomedicine has been used to enhance the immunotherapy in lung cancer. In this review article, we described the association between TME and immunotherapy in lung cancer. We also highlighted the importance of TME in immunotherapy in lung cancer. Moreover, we discussed how nanoparticles are involved in regulation of TME to improve the efficacy of immunotherapy, including Nanomedicine SGT-53, AZD1080, Nanomodulator NRF2, Cisplatin nanoparticles, Au@PG, DPAICP@ME, SPIO NP@M-P, NBTXR3 nanoparticles, ARAC nanoparticles, Nano-DOX, MS NPs, Nab-paclitaxel, GNPs-hPD-L1 siRNA. Furthermore, we concluded that targeting TME by nanoparticles could be helpful to overcome resistance to PD-1/PD-L1 blockade in lung cancer.

## Introduction

Lung cancer is one of the common malignant cancers worldwide (Lee and Kazerooni, 2022). There are about 236,740 new cases of lung cancer and about 130,180 deaths from this disease in the United States (Siegel et al., 2022). In the United States, the 5-year relative survival rates for lung cancer is 22% (Siegel et al., 2022). Cigarette smoking is one key reason for lung cancer development. Lung cancer has three types: non-small cell lung cancer (NSCLC, 82%), small cell lung cancer (SCLC, 14%) and unspecified histology (3%) (Miller et al., 2022). Patients with stage I or II lung cancer often undergo surgery, and patients with stage III lung cancer undergo surgery, chemotherapy and/or radiation (Guo et al., 2022a). However, the lung cancer patients often obtain drug resistance during targeted therapy, chemotherapy and radiotherapy (Wang et al., 2022a; Wu and Lin, 2022).

Immune checkpoints belong to the immune system, which prevent an immune reaction to impair healthy cells (Yu et al., 2022a; Johnson et al., 2022; Song et al., 2022). Immune checkpoint proteins often exist on the surface of T immune cells and tumor cells. After immune checkpoint proteins of T cells bind to other partner proteins of tumor cells, T cells are blocked from impairing the tumor cells (Ma et al., 2021; Hassanian et al., 2022; Korman et al., 2022). For example, immune checkpoint protein PD-1 of T cells can bind with PD-L1 of tumor cells, leading to impairment of killing tumor cells in the body (Hu et al., 2021a; Hou et al., 2023). Immune checkpoint blockade (ICB) with PD-1 antibody or PD-L1 antibody can block the binding between PD-1 and PD-L1, and allow the T cells to destroy tumor cells (Hu et al., 2021b; Jiang et al., 2021; Archilla-Ortega et al., 2022). Immune checkpoint inhibitors (ICIs) often impair the interaction between checkpoint proteins (such as PD-1) and their partner proteins (PD-L1), which allows the T cells to eradicate tumor cells (Havel et al., 2019). For example, one ICI ipilimumab blocks CTLA-4, pembrolizumab and nivolumab blocks PD-1, and atezolizumab, avelumab and durvalumab blocks PD-L1 (Huang and Zappasodi, 2022; Tison et al., 2022). Therefore, ICI therapy has been used in various cancer types, including lung cancer (Fang and Su, 2022; Hao et al., 2022; Mussafi et al., 2022; Punekar et al., 2022; Zulfiqar et al., 2022).

Tumor microenvironment (TME) is an integral tumor microenvironment, which is involved in tumorigenesis, malignant progression and drug resistance (Li and Qiao, 2022; Shi et al., 2022). TME includes complex cellular components, such as tumor-associated macrophages (TAMs), T cells and other immunocytes, blood vessels, fibroblast and extracellular matrix (Liu et al., 2022a). TAMs have been revealed to promote tumor initiation and progression *via* promotion of T cell dysfunction, invasive activity, migratory capacity and angiogenesis (Cao et al., 2022). TAMs can regulate tumor immune microenvironment (TIME) and suppress cytotoxic T lymphocyte (CTL) reaction, resulting in impeding activity of ICIs (Petroni et al., 2022).

Recently, nanomedicine has been widely used to enhance the immunotherapy in lung cancer (Cheng and Santos, 2022). In this review article, we discussed the relationship between TME and immunotherapy in lung cancer. Moreover, we discussed how nanoparticles are involved in regulation of TME to improve the efficacy of immunotherapy. Furthermore, we verified that nanoparticles might target TME to improve immunotherapy efficacy and overcome anti-PD1/PD-L1 therapeutic resistance in lung cancer (Table 1).

**TABLE 1:** Nanoparticles in regulation of TME and immunotherapy in lung cancer.

Nanoparticles	Functions	Immunotherapy	Ref.
SGT-53	Promotion of tumor immunogenicity, enhancement of innate and adaptive immune responses and inhibits immunosuppression	Sensitizes the efficacy of anti-PD1 antibody treatment	Kim et al. (2018)
AZD1080	Enhances delivery to tumor sites	Reduces expression of PD1 and releases CD8^+^ T cells	Allen et al. (2021)
ZVI-NP	Enhances anti-tumor immunity *via* reduction of Treg cells and promotion of M2 macrophages to M1.	Repression of PD1 and CTLA4 in CD8^+^ T cells, reduction of PD-L1 expression	Hsieh et al. (2021)
Cisplatin nanoparticle	Sustained upregulation of PD-L1 expression levels	Promotes the treatment efficacy of anti-PD1/PD-L1 blockade	Shen et al. (2021)
Au@PG nanoparticles	Make tumor remodeling from a cold TME to a hot TME, contributing to tumor suppression and cytotoxic T cell response promotion.	Plus anti-PD1 therapy enhanced tumor suppression and immunosuppression	Su et al. (2022)
DPAICP@ME	Elevates the p53 activation, causes T cell activation	Augments anti-PD1 immunotherapy	He et al. (2022)
SPIO NP@M-P	Maintains the longer half-life of the PD-L1 inhibitory peptides, leading to reactivation of T cells and inhibition of tumor growth	Targets TME for improving immunotherapy	Meng et al. (2021)
NBTXR3 nanoparticles	Modulated the TIME *via* regulation of the T cell receptor repertoire, elevating CD8^+^ T cells and activation of immune signaling pathways	Overcomes anti-PD1 resistance	Hu et al. (2021c)
ARAC	Decreases effective concentrations of volasertib and PD-L1 antibody *via* modulation of CD8^+^ T cells.	Increases the effect of PD-L1 inhibitors	Reda et al. (2022)
Nano-DOX	Enhances immunogenicity of tumor cells and reactivates the TAM into M1 phenotype *via* regulation of RAGE/NF-kappa B.	Induction of PD-L1 in the tumor cells and PD-1/PD-L1 in the TAMs. Improves immunotherapy.	Xu et al. (2021)
MS NPs	Retards tumor metastasis *via* increasing immune surveillance and modifying the extracellular matrix	Improves immunotherapy *via* suppression of PD-L1 expression level by metformin.	Cai et al. (2021)
GNPs-hPD-L1 siRNA)	Inhibit the expression of PD-L1 and serve as photothermal agent for theranostic functions	Improves immunotherapy	Liu et al. (2019)

## TME and immunotherapy

TME has been identified to be associated with lung cancer development and progression (Zhao et al., 2022a; Liu et al., 2022b; Madeddu et al., 2022; Mansouri et al., 2022; Peters et al., 2022). TIME is associated with molecular heterogeneity of immunotherapy efficacy in NSCLC patients (Graves et al., 2010; Jin et al., 2020; Belluomini et al., 2021; Genova et al., 2021). In this section, we discuss the association of TME and immunotherapy.

### Signaling pathways and TME in immunotherapy

Cellular signaling pathways play a prominent role in regulation of TME in lung cancer. For example, depletion of Periostin modulated TME and suppressed bone metastasis *via* repressing integrin signaling pathway in lung cancer (Che et al., 2017). Vav1 was found to modulate TME in Ras-driven lung cancer (Shalom et al., 2022). Vav1 instigated TME by fibrosis enhancement and reduction in tumor infiltrating macrophage, which was due to cross-talking with colony-stimulating factor 1 (CSF1) pathway, favoring lung cancer growth (Sebban et al., 2014). Blockade of IL6 regulated TME and limited the lung cancer development and progression *via* inactivation of STAT3 pathway (Caetano et al., 2016). Indoleamine 2,3-dioxygenase (IDO) pathway governed antitumor immunity *via* metabolic reprogramming of immune cells in TME through regulating AMPK pathway in lung cancer (Schafer et al., 2016). TRAF4 facilitated lung cancer malignant aggressiveness by regulating TME in normal fibroblasts *via* NF-κB pathway-induced ICAM1 upregulation (Kim et al., 2017). One study showed that low dose of IFNγ endowed tumor stemness in TME of NSCLC *via* regulation of ICAM1/PI3K/AKT/Notch-1 pathway, whereas high dose of IFNγ induced apoptosis of NSCLC cells *via* activation of JAK1/STAT1/caspase pathway (Song et al., 2019). Fibronectin in TME activated inflammatory response *via* regulation of TLR4/NF-κB signaling pathway in lung cancer cells (Cho et al., 2020).

Cyclophosphamide, a cytotoxic agent for cancer treatment, modulated TME *via* TGF-β signaling pathway in a mouse model of Lewis lung cancer (Zhong et al., 2020). Low dose cyclophosphamide increased CD4 + /CD8 + T cells and reduced Treg cells as well as reduced myofibroblasts, which was accompanied with upregulation of E-cadherin and downregulation of N-cadherin (Zhong et al., 2020). Dietary restriction attenuated tumor growth and improved TME and inhibited angiogenesis in NSCLC xenografts through regulation of PI3K/AKT and NF-κB/COX2/iNOS pathways (Lin et al., 2013). The diffusible gas carbon monoxide altered TME and impeded tumor growth *via* regulating MAPK/Erk1/2 pathway, Notch-1 pathway, HO-1 and CD86 expressions in lung cancer (Nemeth et al., 2016). Neobavaisoflavone nanoemulsion was reported to block tumor progression *via* targeting TME of lung cancer through repressing TGF-β/SMADs pathway (Ye et al., 2020). A small nucleolar RNA SNORA38B was reported to promote oncogenesis and reduce immunotherapy effects *via* remodeling the TME through targeting GAB2/AKT/mTOR signaling pathway (Zhuo et al., 2022). SNORA38B recruited the CD4+FOXP3 + Treg cells in TME, contributing to poorer immune efficacy in NSCLC (Zhuo et al., 2022). Evidence has suggested that simultaneous targeting of tumor-initiating cells and signaling pathways in the TME led to effective immunotherapy in lung cancer (Dubinett and Sharma, 2009).

### EGFR and ALK mutations affect TME in immunotherapy

EGFR-mutant tumors and ALK-rearranged tumors exhibited a poor response to anti-PD-1/PD-L1 immunotherapy (Jin et al., 2020). TIME was associated with oncogenic manners in NSCLC patients because KRAS mutations and EGFR L858R mutation play a critical role in inflammatory response and immune resistance in tumor microenvironment (Jin et al., 2020). In addition, EGFR-mutant and ALK-rearranged tumors displayed more resting memory CD4^+^ T cells and less CD8^+^ T cells and activated memory CD4^+^ T cells (Jin et al., 2020). Another study also confirmed that targeting the PD-1/PD-L1 did not benefit the EGFR-mutant or ALK-translocated NSCLC patients (Bylicki et al., 2017).

One study reported that CD25^+^; CD4^+^ T cells with high expression of PD-L1 (PD-L1 high Treg) were increased in TME of NSCLC patients (Wu et al., 2018). PD-L1 high Treg was positively linked to PD-1 + CD8 in Treg in NSCLC. Moreover, PD-1/PD-L1 pathway promoted the effect of TILs by anti-PD-1/PD-L1 immunotherapy (Wu et al., 2018). These NSCLC patients with PD-L1 expression exhibited a better prognosis. Hence, the density of PD-L1 +; CD4^+^; CD25^+^ Tregs in TME could be a diagnostic predictor and immunotherapy response markers in NSCLC (Wu et al., 2018). Another study implied that lymphocyte activing 3 (LAG-3) expression was associated with TILs, PD-1, PD-L1 and survival in NSCLC (He et al., 2017). LAG-3 was expressed on TILs in NSCLC patients’ tumor tissues, which was associated with the expression of PD-1 and PD-L1 in NSCLC. NSCLC patients with LAG-3 positivity or both PD-L1 and LAG-3 positivity had an early recurrence and poor prognosis (He et al., 2017).

Low expression of PD-1 in cytotoxic CD8^+^ TILs indicated a privileged TIME in NSCLC, which suggested a predictive and prognostic values (Mazzaschi et al., 2018). NSCLC patients with low PD-1 expression in CD8^+^ cells after nivolumab treatment displayed a prolonged survival, indicating that CD8^+^; PD-1 low was a prediction factor for response to immunotherapy in NSCLC (Mazzaschi et al., 2018). Similarly, one tissue microarray showed that CD8^+^ cells, especially PD-L1 negative tumors lacking PD-1 + TILs had a big prognostic value. PD-L1 positive tumors with CD8^+^ lymphocytes can promote the survival in NSCLC (Munari et al., 2021). In addition, PD-L1 overexpression had an unfavorable prognosis and high CD8^+^ TILs had a favorable prognosis in NSCLC patients (Rashed et al., 2017). Zhang et al. reported that PD-L1 plus CD8^+^ TILs established an immunosuppressive TME with high mutation burden in NSCLC (Zhang et al., 2021). NSCLC patients with high PD-L1+; CD8^+^ TILs had a better response to the anti-PD-1 immunotherapy (Zhang et al., 2021). Consistently, differential TIME determined immunotherapy efficacy in NSCLC patients with advanced stages (Shirasawa et al., 2021).

Four types of groups from 228 NSCLC patients were identified: type I, 73 patients with PD-L1 high/TIL high; type II: 70 cases with PD-L1 low/TIL low; type III: 37 patients with PD-L1 high/TIL low; type IV: 48 cases with PD-L1 low/TIL high. Each type patients had a different survival: Type I tumors had good prognosis compared with type III tumors (Shirasawa et al., 2021). Yang et al. also observed that PD-L1 expression in combination with CD8^+^ TILs showed a prognostic value in patients with NSCLC after surgery (Yang et al., 2018). A retrospective study defined that PD-L1 expression plus CD8^+^ TILs density is useful for prediction of disease-free survival in lung squamous cell carcinoma after surgery (Cheng et al., 2022). In addition, PD-L1 expression and TIL infiltration appeared in brain metastasis of small cell lung cancer. PD-L1 TILs and CD45RO+ memory T cells were linked to favorable survival (Berghoff et al., 2016). Lung cancer patients with intracranial resection of brain metastases had a better outcomes if the patients had PD-L1 positivity and a high intraepithelial CD8^+^ T cell infiltration (Li et al., 2022a). Zhou et al. discovered that paired primary NSCLC and brain metastatic lesions in NSCLC have a difference for PD-L1 expression and CD8^+^ TILs (Zhou et al., 2018). There were a fewer CD8^+^ TILs in brain metastatic tissues *versus* primary lung tumor samples, which was associated with shorter overall survival *versus* high CD8^+^ TILs density (Zhou et al., 2018). However, Batur et al. found that PD-L1 expression and CD8^+^ TIL intensity had a concordance between brain metastases and NSCLC (Batur et al., 2020). Hence, further investigation is required to determine the role of PD-L1, CD8^+^ TIL and TIME in immunotherapy of NSCLC patients (Vilarino et al., 2020). Notably, seven randomized controlled trials had uncovered that PD-1/PD-L1 inhibitors exhibited a treatment efficacy in brain metastases of NSCLC, reducing risk of disease progression and death in NSCLC patients with brain metastases (Li et al., 2022b). Strikingly, TME, including spatial and temporal discordance of TILs and PD-L1 expression, was discovered between lung primary lesions and brain metastases in lung cancer (Mansfield et al., 2016). Together, PD-1, PD-L1 expression and TIL status can predict the response of anti-PD-1/PD-L1 treatment in NSCLC (Nakagawa and Kawakami, 2022).

## TME and CCRT in NSCLC patients

TME has been known to affect concurrent chemoradiation therapy (CCRT) in NSCLC patients (Shirasawa et al., 2020). Next, we summarize how TME regulates CCRT *via* CD8^+^ TILs and PD-L1 in NSCLC. A retrospective research showed that chemoradiation therapy can change the expression of PD-L1 and CD8^+^ TILs (Choe et al., 2019). NSCLC patients with PD-L1 expression had a short survival after concurrent chemoradiation therapy (CCRT). Patients with an upregulation of CD8^+^ TILs after CCRT displayed a longer overall survival (Choe et al., 2019). Moreover, Tokito et al. defined predictive relevance of PD-L1 plus CD8^+^ TIL density in patients with stage III NSCLC after CCRT (Tokito et al., 2016). PD-L1+/CD8 low patients had the worst overall survival, whereas PD-L1-/CD8 high patients had the best prognosis (Tokito et al., 2016). PD-L1+ tumor cells were reduced after CCRT in NSCLC patients. Modulation of PD-L1 expression was linked to prognosis in locally advanced NSCLC patients after CCRT (Fujimoto et al., 2017). Similarly, evidence showed that PD-L1 expression was linked to high tumor grade and low density of CD8 TILs (El-Guindy et al., 2018). PD-L1-/CD8 high patients had a good overall survival, while PD-L1+/CD8 low patients often had advantage tumor stage and the poorest overall survival (El-Guindy et al., 2018).

One clinical trial also confirmed the alteration in tumoral PD-L1 expression and stromal CD8^+^ TILs in NSCLC patients after CCRT (Yoneda et al., 2019). PD-L1 expression was increased in NSCLC patients after CCRT, and stromal CD8^+^ TIL was elevated after CCRT. Higher CD8^+^ TILs supported a favorable prognosis. CCRT-induced PD-L1 expression in NSCLC after CCRT suggested that PD-L1 blockade in combination with CCRT is necessary to improve survival in NSCLC (Yoneda et al., 2019). PD-L1-/CD8 low NSCLC patients after CCRT had the longest overall survival, while PD-L1+/CD8 low patients with locally advanced NSCLC after CCRT had the shortest overall survival (Gennen et al., 2020). One study also discovered that PD-L1 expression was changed after CCRT, the density of CD8^+^ TILs was upregulated after CCRT. Moreover, locally advanced NSCLC patients after CCRT had a good response to anti-PD-1/PD-L1 therapy (Shirasawa et al., 2020).

## In lung cancer mouse models

To better understand the role of TME in PD-1 blockade resistance in lung cancer, one group used genetically engineered lung cancer mouse models (Martinez-Usatorre et al., 2021). The mouse models included Kras^G12D/+^; p53^−/−^ (KP) mice, Kras^G12D/+^; p53^−/−^; Msh2^-/-^ (KPM) mice and Kras^G12D/+^; p53^−/−^; ovalbumin (KPO) mice. Blockade of ANGPT2 and VEGFA by a bispecific antibody A2V retarded progression and metastasis of KP lung tumors *via* development of a favorable TME and modulation of the immune cell composition of KP tumors (Martinez-Usatorre et al., 2021). Specifically, A2V treatment was correlated with reprogramming of the TIME through increased T cells and decreased TAMs (Martinez-Usatorre et al., 2021). Interestingly, inhibition of PD-1 by its antibody failed to improve tumor response to A2V treatment in KP mice. However, the PD-1 antibody affected PD-1^+^ Tregs in KP tumors (Martinez-Usatorre et al., 2021). Moreover, TAMs interacted with Tregs in KP tumors. Furthermore, CSF1R suppression in combination with cisplatin inhibited TAMs and enhanced the efficacy of anti-angiogenic immunotherapy (Martinez-Usatorre et al., 2021). Another study used the Kras^LSL−G12D^/+Tp53^fl/fl^ (KP) and the Kras^LSL−G12D^/+Lkb1^fl/fl^ (KL) NSCLC mouse models to determine the immunotherapy efficacy and TME of NSCLC (Zhang et al., 2020a). This work showed that CCL7 enhanced anti-PD-1 therapy in these mouse models *via* promoting conventional DC 1 into TME, leading to T cell expansion. In NSCLC tissues, CCL7 expression was elevated, and it was associated with conventional DC 1 infiltration and overall survival in NSCLC patients (Zhang et al., 2020a). In KP mouse model, depletion of CCL7 destroyed the conventional DC 1 infiltration in the TME and promoted expansion of CD4^+^ and CD8^+^ T cells in TME, resulting in tumor development. Upregulation of CCL7 in lungs retarded tumor development and increased the survival of KP and KL mice. Thus, CCL7 could act a biomarker for anti-PD-1 therapy of NSCLC (Zhang et al., 2020a).

## Nanomedicine

Nanomedicine is a novel unique branch of medicine using nano-size technology for exploration of underlying mechanisms of disease development and progression and for the prevention, diagnosis and therapy of various diseases (de Lazaro and Mooney, 2021; Stater et al., 2021). Nanomedicine has been used for cancer diagnosis and therapy *via* merging the physical, biological, chemical and digital technologies together (Ahmad et al., 2023; Li et al., 2023). In recent years, nanomedicine has exhibited a potent effect in immunotherapy for cancer patients (Irvine and Dane, 2020; Guo et al., 2022b; Wang et al., 2022b; Yang et al., 2023).

### Nanomedicine enhances immunotherapy in lung cancer

Evidence has suggested that nanomedicine could enhance immunotherapy in NSCLC (Seshadri and Ramamurthi, 2018; Garcia-Fernandez et al., 2020; Pu et al., 2022). For example, TME component-targeted nanomedicine delivery facilitated the efficacy of immune checkpoint inhibitors (Kim et al., 2021). In addition, nanoparticle-based ICI therapy elevated the local dose of ICIs and reduced the side effects of ICIs, leading to boosting the anti-tumor immunity in several types of cancers, including lung cancer (Sanaei et al., 2021).

### Nanomedicine SGT-53 enhances anti-PD1 antibody immunotherapy

One group designed SGT-53, a nanomedicine carrying a p53 plasmid, to detect whether SGT-53 can augment immune checkpoint inhibitor therapy (Kim et al., 2018). This group used three mouse models, including a glioblastoma, a NSCLC and a breast cancer. SGT-53 sensitized the efficacy of anti-PD1 antibody treatment *via* promotion of tumor immunogenicity, enhancement of innate and adaptive immune responses and inhibited immunosuppression in a breast tumor model (Kim et al., 2018). STG-53 in combination of an anti-PD1 antibody was stronger than each agent individually in suppression of tumor growth and metastasis. STG-53 blocked fatal xenogeneic hypersensitivity after anti-PD1 therapy in breast cancer model (Kim et al., 2018). This work indicated that nanomedicine SGT-53 restored p53 biological function and caused anti-tumor immunity to increase sensitization of anti-PD1 treatment in human cancer.

### Nanoparticle AZD1080 enhances delivery to tumor sites

One study used the remote loading of GSK3 inhibitor AZD1080 into nanoparticles coated with a lipid bilayer. Intravenous injection of AZD1080 nanoparticles enhanced biodistribution and drug delivery to cancer site and reduced the expression of PD1 and released CD8^+^ T cells (Allen et al., 2021). Encapsulated AZD1080 reduced tumor growth in CT26 colorectal tumor, KPC pancreatic cancer and LLC lung cancer models without treatment toxicity. Hence, nano drug delivery of AZD1080 could be used in combination with immunotherapy or chemotherapy in human cancer (Allen et al., 2021).

### Nanomodulator NRF2 induces an immunostimulatory TME

It has been reported that zero-valent-iron nanoparticle (ZVI-NP) triggered anticancer immunity and cancer-specific cytotoxicity in lung cancer (Hsieh et al., 2021). ZVI-NP induced ferroptotic death *via* regulation of lipid peroxidation, mitochondria dysfunction and ROS in lung tumor cells. Furthermore, β-TrCP-induced NRF2 destruction *via* AMPK/mTOR pathway was increased in this kind of ferroptosis. ZVI-NP suppressed angiogenesis-associated genes and reduced the self-renewal capacity. ZVI-NP enhanced anti-tumor immunity *via* reduction of Treg cells and promotion of M2 macrophages to M1, and repression of PD1 and CTLA4 in CD8^+^ T cells, as well as reduction of PD-L1 expression in tumor cells. Strikingly, ZVI-NP mainly stayed in lung tissues and tumor sites, resulting in inhibition of tumor metastasis and growth (Hsieh et al., 2021). Taken together, NRF2 nanomodulator stimulated lung cancer ferroptosis and maintained an immunostimulatory TME.

### Cisplatin nanoparticles sensitize PD1/PD-L1 inhibitors

Cisplatin nanoparticles improved the PD1/PD-L1 inhibitor therapeutic outcomes because cisplatin nanoparticles increased the expression of PD-L1 levels (Shen et al., 2021). Cisplatin nanoparticles in combination with PD1/PD-L1 inhibitors, BMS-202 and anti-PD1 antibody, caused a superior inhibition of tumor growth. Cisplatin nanoparticles plus anti-PD1 antibody displayed a stronger tumor inhibition than cisplatin plus anti-PD1 antibody in the LLC tumor model. Altogether, cisplatin nanoparticle promoted the treatment efficacy of anti-PD1/PD-L1 blockade through sustained upregulation of PD-L1 expression levels (Shen et al., 2021).

### Au@PG nanoparticles improve immunotherapy

Polyaniline-based glyco-condensation on Au nanoparticles have been found to promote immunotherapy in lung cancer (Su et al., 2022). Au@PG nanoparticles can make tumor remodeling from a cold TME to a hot TME, contributing to tumor suppression and cytotoxic T cell response promotion. Au@PG nanoparticles plus anti-PD1 therapy enhanced tumor suppression and immunosuppression and improved cytokine secretion (Su et al., 2022). Moreover, the size of Au@PG nanoparticles made a decision for the switch from M2 to M1 macrophages: the smaller Au@PG nanoparticles exhibited better functions than larger ones. Au@PG nanoparticles caused endoplasmic reticulum stress and spleen tyrosine kinase activation and macrophage polarization in lung cancer (Su et al., 2022).

### DPAICP@ME augments anti-PD1 immunotherapy

A chiral-peptide supramolecular (DPAICP) interacting with the membrane from milk-derived extracellular vesicles (ME) was constructed (He et al., 2022). DPAICP@ME was found to be stable in blood circulation after gastrointestinal absorption, and displayed tumor accumulation by oral medication. Oral DPAICP@ME elevated the p53 activation for treating cancer in LLC lung cancer orthotopic model and PDOX mice of colon cancer and B16F10 homograft melanoma model (He et al., 2022). Oral DPAICP@ME caused T cell activation and led to enhancement of anti-PD1 immunotherapy. Hence, DPAICP@ME could boost the anti-PD1 immunotherapy in human cancer (He et al., 2022).

### SPIO NP@M-P targets TME for immunotherapy

Superparamagnetic iron oxide nanoparticles (SPIO NPs) were combined with lung cancer H460 cell membranes, PD-L1 inhibitory peptide (TPP1) and MMP2 substrate peptide (PLGLLG), which were named as SPIO NP@M-P (Meng et al., 2021). The TPP1 peptide with homotypic effect of cancer cell membrane was entered to the TME and digested by MMP2 enzyme. Therefore, SPIO NP@M-P maintained the longer half-life of the PD-L1 inhibitory peptides, leading to reactivation of T cells and inhibition of tumor growth (Meng et al., 2021). SPIO NP@M-P could be a useful platform for cancer therapy and tumor diagnosis.

### NBTXR3 nanoparticles overcomes anti-PD1 resistance

To overcome anti-PD1 resistance in lung cancer, one group combined radiation with NBTXR3 nanoparticles and anti-PD1 therapy (Hu et al., 2021c). This group reported that the triple combination (anti-PD1, localized radiation and NBTXR3) reduced growth of irradiated and unirradiated tumors in 344SQP anti-PD1-sensitive lung cancer cells and 344SQR anti-PD1-resistant lung cancer cells (Hu et al., 2021c). Moreover, NBTXR3 modulated the TIME of unirradiated tumors *via* regulation of the T cell receptor repertoire, elevating CD8^+^ T cells and activation of immune signaling pathways in 344SQR tumor model. NBTXR3 nanoparticles could be helpful for treating metastatic lung cancer patients regardless of immunotherapeutic resistance or sensitivity (Hu et al., 2021c). Later, this group combined NBTXR3 with three inhibitors of checkpoint receptors: PD1, TIGIT and LAG3 (Hu et al., 2022). The nanoparticle-involved combination treatment reduced the growth of irradiated and unirradiated tumors due to activation of the immune response and increased immune cells (Hu et al., 2022). Furthermore, a triple-combination therapy includes NBTXR3, high-dose radiation (HDXRT) for primary tumors and low-dose radiation (LDXRT) for a secondary tumor, and ICIs (Hu et al., 2021d). This triple-combination therapy displayed remarkable anticancer activity and improve the survival in mice of anti-PD1-resistant lung cancer. NBTXR3+HDXRT + LDXRT reduced the number of Treg cells and promoted CD8 T cell infiltration. NBTXR3 nanoparticle plus radioimmunotherapy enhance antitumor immune response and promote the survival (Hu et al., 2021d).

### ARAC nanoparticles target PD-L1

A nanoparticle-based treatment named antigen release agent and checkpoint inhibitor (ARAC) was discovered to increase the effect of PD-L1 inhibitors (Reda et al., 2022). ARAC nanoparticle co-delivered PLK1 inhibitor (volasertib) and PD-L1 antibody. Because PLK1 was often upregulated in lung cancer and promoted tumor growth, suppression of PLK1 could reduce cancer growth. ARAC decreased effective concentrations of volasertib and PD-L1 antibody in LLC tumor model *via* modulation of CD8^+^ T cells. ARAC exhibited therapy efficacy in KLN-205 lung tumor model (Reda et al., 2022).

### Nano-DOX improve immunotherapy

Nanodiamond-doxorubicin conjugates (Nano-DOX) in combination with anti-PD-L1 agent BMS-1 synergistically enhanced tumor suppression (Xu et al., 2021). Nano-DOX enhanced immunogenicity of tumor cells and reactivated the TAM into M1 phenotype *via* regulation of RAGE/NF-κB pathway and induction of PD-L1 in the tumor cells and PD-1/PD-L1 in the TAMs. Nano-DOX increased the cytokine HMGB1 *via* targeting RAGE/NF-κB pathway (Xu et al., 2021). BMS-1 promoted M1 activation of TAMs that was induced by Nano-DOX *via* reducing PD-L1 in the TAMs and impairing the interaction between PD1 and PD-L1, contributing to inhibition of tumor growth due to killing tumor cells. Nano-DOX enhanced BMS-1 efficacy on tumor growth in a TAM-mediated manner (Xu et al., 2021).

### MS NPs enhance chemo-immunotherapy

A nanodrug (MS NPs) was developed to combine immunoadjuvant metformin with 7-ethyl-10-hydroxycamptothecin (SN38) *via* electrostatic interactions (Cai et al., 2021). MS NPs improved immunotherapy *via* suppression of PD-L1 expression level by metformin. MS NPs were also found to retard tumor metastasis *via* increasing immune surveillance and modifying the extracellular matrix (Cai et al., 2021). Importantly, MS NPs increased mouse survival with no obvious toxicity. MS NPs increased the efficacy of ICIs, indicating that MS NPs could open a new window for the development of novel anti-PD1/PD-L1 therapy (Cai et al., 2021).

### Other nanoparticles improve immunotherapy

One group designed nanoarchitecture using anti-PD-L1 antibody and magnetic-nanoparticle-attached YFCD for the separation and identification of PD-L1-expressing exosomes (Pramanik et al., 2022). Different lung cancer cell lines had a different amount of PD-L1+ exosomes. H460 lung cancer cells expressed huge PD-L1+ exosomes, and A549 cancer cells expressed low PD-L1+ exosomes, while normal skin HaCaT cells did not express PD-L1+ exosomes (Pramanik et al., 2022). The nanoarchitectures with YFCDs and anti-PD-L1 antibody separated and tracked PD-L1+ exosomes, suggesting that this nanoarchitecture could be used for clinical application to analyze PD-L1+ exosomes, which can help immunotherapy (Pramanik et al., 2022). Nanoparticle albumin-bound (Nab)-paclitaxel had improved the survival of older patients with stage IV NSCLC after disease progression with platinum-based doublet chemotherapy (Weiss et al., 2020). A gold nanoprism-assisted human PD-L1 siRNA (GNPs-hPD-L1 siRNA) was designed to inhibit the expression of PD-L1 and serve as photothermal agent for theranostic functions in lung cancer (Liu et al., 2019).

## Conclusion

**FIGURE 1:**
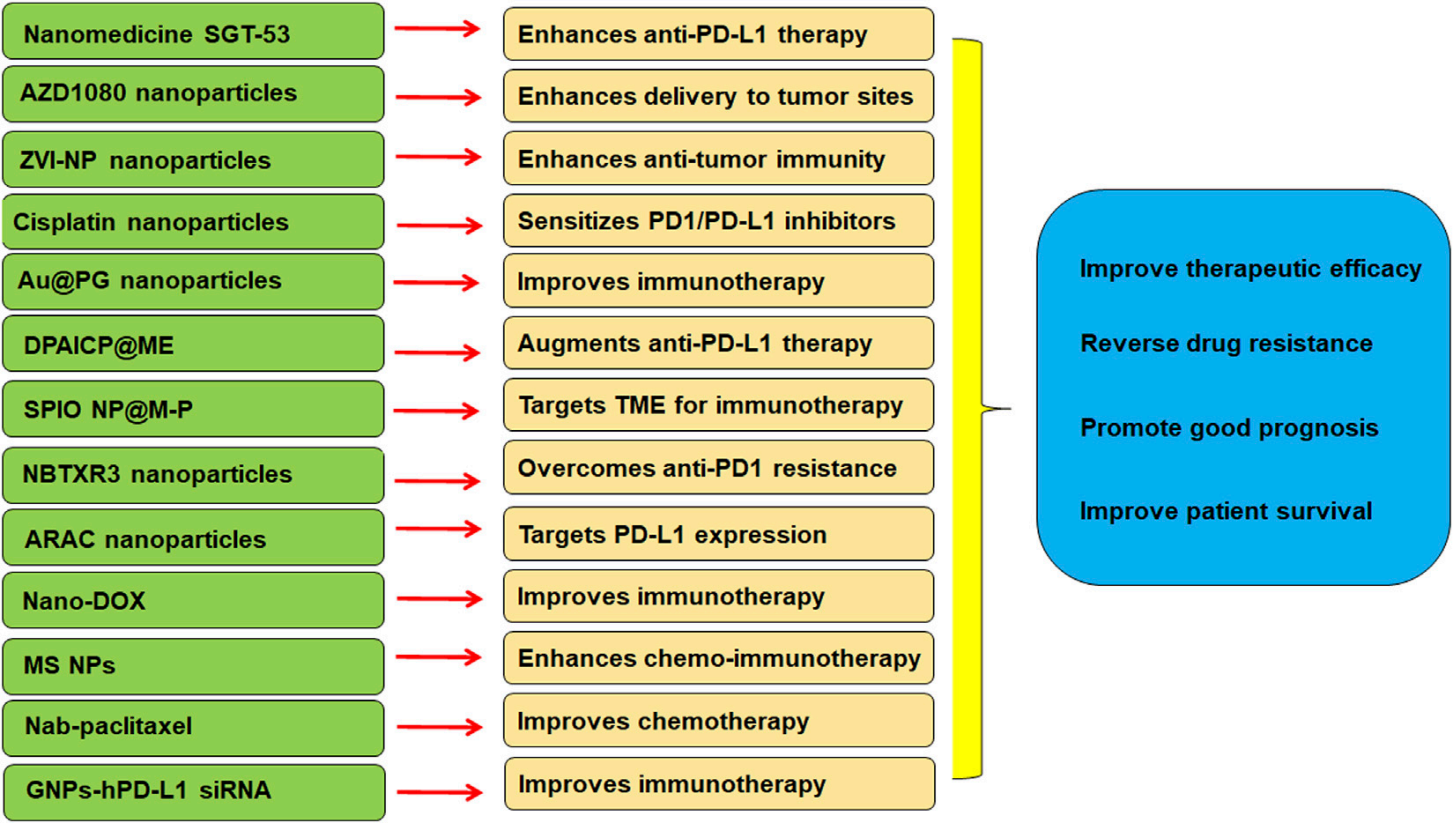
The role of nanoparticles in regulation of TME and immunotherapy in lung cancer.

In conclusion, there is a close association between TME and immunotherapy in lung cancer. Targeting TME could be a strategy for overcoming resistance to PD-1/PD-L1 blockade in lung cancer. ICIs have demonstrated the therapeutic benefits in NSCLC. But ICIs have several side effects, such as ICI-related pneumonitis (Conroy and Naidoo, 2022; Hao et al., 2022). Altogether, nanoparticle-based ICI therapy can boost the anti-tumor immunity in lung cancer (Figure 1).

Several issues need to be highlighted in lung cancer therapy. For example, mRNA vaccine development to recognize immune-associated tumor antigens and immune subtypes in lung cancer (Wang et al., 2021; Zhao et al., 2022b; Zhao et al., 2022c; Xu et al., 2022; Zhou et al., 2022). Similar mRNA vaccines have been reported in other cancers, such as glioma and melanoma (Zhong et al., 2021; Sittplangkoon et al., 2022). Moreover, lipid nanoparticle-based mRNA cancer vaccines displayed advantages in cancer therapy ((Persano et al., 2017; Chen et al., 2022a; Huang et al., 2022a)). In addition, non-coding RNAs have been revealed to participate in tumorigenesis in various cancer types ((Chen et al., 2022b; Yu et al., 2022b; Liu and Shang, 2022)). RNA nanotechnology has emerged in cancer therapy in recent years [(Guo, 2010), (Huang et al., 2022b)]. It is important to use nanomedicine to treat cancer cells *via* modulation of non-coding RNAs in lung cancer.

Recently, there is an association between lung cancer, COVID-19 and vaccines [(Trivanovic et al., 2022), (Mao et al., 2021)]. Lung cancer patients have an increased risk from COVID-19 infection and exhibit poor outcomes [(Oldani et al., 2022), (Aramini et al., 2022)]. In addition, genomics, transcriptomics, proteomics, lipidomics and metabolomics are important to be used to determine the mechanisms of disease development and carcinogenesis [(Zhou et al., 2019; Zhang et al., 2020b; Boys et al., 2022)]. The role of multi-omics has been described in lung cancer early detection and therapy [(Abbasian et al., 2022), (Ling et al., 2022)]. The multi-omics should be applied for exploration of TME and immunotherapy in lung cancer to overcome the immunotherapy resistance. Lastly, it must be mentioned that nanomaterials could have side-effects, such as toxicity, and delivery problems. The degradation byproducts from the nanomaterials could have toxicity for host cells. Hence, it is critical to solve these disadvantages of nanomaterials when they are used for cancer therapy to improve immunotherapeutic efficacy in lung cancer.

## Abbreviations

ALK, anaplastic lymphoma kinase; EGFR, epidermal growth factor receptor; GAB2, GRB2-associated-binding protein 2; ICAM1, intercellular adhesion molecule-1; ICIs, immune checkpoint inhibitors; KRAS, Kirsten rat sarcoma; mTOR, mammalian target of rapamycin; NSCLC, non-small cell lung carcinoma; OS, overall survival; PD-1, programmed cell death-1; PD-L1, programmed cell death ligand-1; ROS1, receptor tyrosine kinase c-ros oncogene 1; TME, tumor microenvironment; TIME, tumor immune microenvironment; YFCD, yellow fluorescent carbon dot.
